# Tumor Microenvironment: Key Players in Triple Negative Breast Cancer Immunomodulation

**DOI:** 10.3390/cancers13133357

**Published:** 2021-07-04

**Authors:** Hongmei Zheng, Sumit Siddharth, Sheetal Parida, Xinhong Wu, Dipali Sharma

**Affiliations:** 1 Hubei Provincial Clinical Research Center for Breast Cancer, Department of Breast Surgery, Hubei Cancer Hospital, Tongji Medical College, Huazhong University of Science and Technology, Wuhan 430079, China; 2 The Sidney Kimmel Comprehensive Cancer Center, Department of Oncology, Johns Hopkins University School of Medicine, Baltimore, MD 21218, USA; ssiddha2@jhmi.edu (S.S.); sparida1@jhu.edu (S.P.); dsharma7@jhmi.edu (D.S.)

**Keywords:** triple negative breast cancer, tumor microenvironment, immunomodulation

## Abstract

**Simple Summary:**

The tumor microenvironment (TME) is a complicated network composed of various cells, signaling molecules, and extra cellular matrix. TME plays a crucial role in triple negative breast cancer (TNBC) immunomodulation and tumor progression, paradoxically, acting as an immunosuppressive as well as immunoreactive factor. Research regarding tumor immune microenvironment has contributed to a better understanding of TNBC subtype classification. Shall we treat patients precisely according to specific subtype classification? Moving beyond traditional chemotherapy, multiple clinical trials have recently implied the potential benefits of immunotherapy combined with chemotherapy. In this review, we aimed to elucidate the paradoxical role of TME in TNBC immunomodulation, summarize the subtype classification methods for TNBC, and explore the synergistic mechanism of chemotherapy plus immunotherapy. Our study may provide a new direction for the development of combined treatment strategies for TNBC.

**Abstract:**

Triple negative breast cancer (TNBC) is a heterogeneous disease and is highly related to immunomodulation. As we know, the most effective approach to treat TNBC so far is still chemotherapy. Chemotherapy can induce immunogenic cell death, release of damage-associated molecular patterns (DAMPs), and tumor microenvironment (TME) remodeling; therefore, it will be interesting to investigate the relationship between chemotherapy-induced TME changes and TNBC immunomodulation. In this review, we focus on the immunosuppressive and immunoreactive role of TME in TNBC immunomodulation and the contribution of TME constituents to TNBC subtype classification. Further, we also discuss the role of chemotherapy-induced TME remodeling in modulating TNBC immune response and tumor progression with emphasis on DAMPs-associated molecules including high mobility group box1 (HMGB1), exosomes, and sphingosine-1-phosphate receptor 1 (S1PR1), which may provide us with new clues to explore effective combined treatment options for TNBC.

## 1. Introduction

Triple negative breast cancer (TNBC), characterized by the absence of estrogen receptor (ER), progesterone receptor (PR), and human epidermal growth factor receptor 2 (HER2) expression, comprises 10–20% of all breast cancers [1]. Owing to the lack of ER/PR/Her2 protein expression/amplification, TNBCs do not respond to existing endocrine and Her2-targeted therapies and exhibit poor prognosis [2]. It has been proposed that TNBCs with a higher involvement of immune cells termed as ‘hot tumors’ have better prognosis and a greater response to immunotherapy while TNBCs with a lower involvement of immune cells termed as ‘cold tumors’ are marked with poor prognosis and poor response to immunotherapy [3]. From this point of view, TNBC patients have been further segregated into different subgroups [4,5,6,7,8]. The tumor microenvironment (TME) is an ensemble of endothelial cells, cells of the immune system, adipocytes, and fibroblasts, in addition to the soluble factors released from all the cellular components (including cancer cells) [9,10]. TME can be classified from different perspectives such as host and non-host origin, cellular origin and constituents [9,11,12,13]. TME presents a complex network that plays a crucial role in TNBC immunomodulation and tumor progression.

Cancer initiation and development is not just a biological process triggered by cancer cells in isolation; in fact, it has to be evaluated along with the complicated TME with an emphasis on the interaction between cancer cells and their surrounding extra-cellular matrix. Indeed, considering alterations in microenvironment as active players during cancer progression brings another dimension of complexity [14]. During TNBC progression, tumor immune microenvironment remodeling including the change of the ratio of immune cells and release of multiple immune inhibitory and reactive cytokines is a critical feature [15,16]. Based on the constituents of TME, TNBCs have been stratified into ‘tumor immune microenvironment (TIME) subtypes’ aiding in predicting outcomes and proposing potential treatments guided by the distinct phenotypes of TNBC [16,17]. Chemotherapy, the foremost treatment for TNBC, could induce immunogenic cell death (ICD) and promote the release of damage-associated molecular patterns (DAMPs) [18] including high mobility group box1 (HMGB1), exosomes and sphingosine-1-phosphate receptor 1 (S1PR1) by damaged or activated cells via the activation of TLR4 signal pathway [19] and stimulate the release of various immune molecules such as TGF-β, IK12p7, and IFN-γ [20].

In this review, we focus on immune TME and summarize its immunosuppressive and immunoreactive roles, discuss constituent immune cells involved in TNBC immunomodulation, and the contribution of TIME in stratification of TNBC. Further, we discuss the role of chemotherapy-induced TME changes in modulating TNBC immune response and tumor progression, with a focus on HMGB1, exosomes, and sphingosine-1-phosphate (S1P)/sphingosine kinase 1 (SPHK1)/S1PR1, an axis whose therapeutic modulation may result in neoteric combination therapy for TNBC patients.

## 2. Two Roles of TME in TNBC Immunomodulation

**Figure 1:**
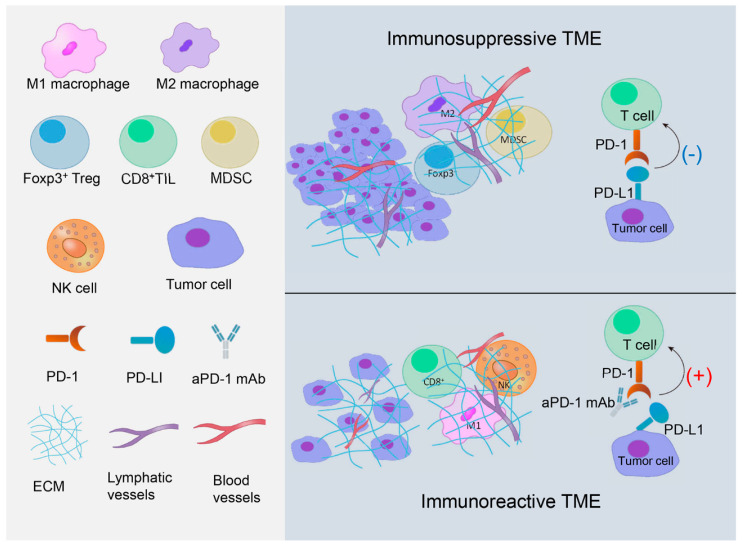
Immunosuppressive and immunoreactive TME. Immunosuppressive tumor microenvironment (TME) is mainly constituted of M2 macrophages, forkhead box P3^+^ (Foxp3^+^) regulatory T lymphocytes (Tregs), myeloid-derived suppressor cells (MDSCs), and PD-1/PD-L1 axis. Immunoreactive TME is mainly constituted of CD8^+^ T cells, natural killer (NK) cells, and M1 macrophages. PD-1/PD-L1 axis becomes immunoreactive in response to anti-PD1 or anti-PD-L1 monoclonal antibody (aPD-1/PD-L1 mAb) owing to the activation of CD8^+^ T cells. (Foxp3, forkhead box P3; Tregs, regulatory T lymphocytes; MDSC, myeloid-derived suppressor cell; NK, natural killer; PD-1, programmed cell death protein-1; PD-L1, programmed death-ligand 1; aPD-1 mAb, anti-PD-1 monoclonal antibody; ECM, extra cellular matrix; TME, tumor microenvironment).

According to the contribution to immune response, the tumor microenvironment (TME) can be classified as immunosuppressive and immunoreactive. Tumor infiltrating lymphocytes (TILs), the major cell types in the microenvironment, are heterogeneous and mainly composed of lymphocytes in tumor nests and tumor stroma. TILs can be classified into several different subtypes, mainly CD3^+^ T cells and CD20^+^ B cells in solid tumors, though CD20^+^ B cell infiltration is relatively less. CD3^+^ T cells include CD8^+^ cytotoxic T lymphocytes (CD8^+^ TILs), CD4^+^ helping T lymphocytes, and Foxp3^+^ regulatory T lymphocytes (Foxp3^+^ Tregs) [21,22]. Different subtypes of TILs take part in immunomodulation with distinct mechanisms and play various roles in breast cancer immunomodulation [22]. Figure 1 pictorially represents immunosuppressive and immunoreactive TMEs (Figure 1).

### 2.1. Immunosuppressive TME in TNBC

#### 2.1.1. PD-1/PD-L1 Axis

Programmed death-ligand 1 (PD-L1) and programmed cell death protein-1 (PD-1) are important negative co-stimulating signaling molecules in immunoglobulin superfamily (IgSF) and play an important role in host immunomodulation [23]. PD-L1 is expressed in many solid tumors including breast cancer and is a negative prognosis indicator [24,25]. PD-1 is expressed in TILs [26]. Theoretically, PD-L1 expression on tumor cells combined with PD-1 expression on TILs play a negative role in immunomodulation, which inhibits the activation of TILs, causing the tumor cell to survive through immune escape.

The TME involves immune suppressing factors to support the progression of tumors which have escaped host immune surveillance [27,28,29,30,31]. Various immune check-point inhibitors have been developed that have shown efficacy in TNBC patients [32,33]. Clinical studies have shown a paradoxical role of PD-L1 regarding its prognostic value in patients with TNBC owing to the heterogeneity of PD-L1 expression in different tumor sites, non-standard detection methods, and distinct antibodies [31,34,35,36,37,38,39,40,41]. In the impassion 130 clinical trial, compared to TNBC patients receiving nab-paclitaxel plus placebo, a better median overall survival (OS) was observed in patients receiving atezolizumab (PD-L1 inhibitor) combined with nab-paclitaxel and most benefit was observed in PD-L1 positive subgroup [42]. However, in a phase 1b clinical trial (ClinicalTrials.gov Identifier: NCT01848834) which evaluated the safety and effectiveness of PD-1 inhibitor (pembrolizumab) in PD-L1 positive TNBC patients, the overall response rate was only 18.5% and the expression level of PD-L1 was not significantly related to the clinical response [43]. These disparate results might be related to multiple TME-related factors that can modulate the therapeutic effects of PD-1/PD-L1 inhibitors in TNBC. Preclinical studies have shown that PD-L1 expression is modulated by multiple signaling pathways including microRNA-200/ZEB1 axis, WNT, loss of PTEN, PI3K, and MUC1-C/MYC/NF-κB axis [31,44,45,46]. Voorwerk and colleagues reported that doxorubicin and cisplatin treatment caused an upregulation of inflammation-related genes JAK-STAT and TNF-α signaling, immune-related genes associated with PD-1/PD-L1, and T cell cytotoxicity pathways. Short-term and low-dose doxorubicin and cisplatin may create an immunoreactive TME and increase the response to PD-1 inhibitor in TNBC [47]. In conclusion, specifically designed clinical trials are needed to interrogate the involvement of various TME-related factors in order to enhance the efficacy of PD-1/PD-L1 inhibitors in TNBC.

#### 2.1.2. Foxp3^+^ Tregs

In TME, different classes of TILs exist, which have shown great prognostic value in patients with TNBC. Regulatory T lymphocytes (Tregs) are a lineage of lymphocytes involved in immunosuppression that are characterized by the expression of the forkhead box P3 (Foxp3) transcription factor [48,49]. Foxp3^+^ Tregs are the major constituent of the TILs in claudin-low TNBC tumors and it has been speculated that the recruitment of Foxp3^+^ Tregs to the TME inhibits an effective anti-tumor immune response of checkpoint inhibitors [50]. Jamiyan and colleagues detected the expression of stromal Foxp3^+^ Tregs in 107 TNBC samples using IHC and found that a low stromal Foxp3^+^ Tregs level was significantly associated with favorable recurrence free survival (RFS) and OS [51]. In contrast, high Foxp3^+^ TILs expression in 43 TNBC tissues by IHC and Foxp3^+^/CD25^+^ TILs were positively correlated with better OS [52]. High densities of intra-tumoral Tregs and CD20^+^ B cells represented a good prognostic panel in TNBCs [53]. However, mRNA expression of Foxp3 by qRT-PCR in 826 breast tumor tissue samples including 84 TNBC samples, was not significantly related to disease free survival (DFS), while none of the markers studied including CD3, CD8, and Foxp3 were of prognostic value for OS [54]. This phenomenon is somewhat explained by a study showing that activation of tumor antigen-specific Tregs in the bone marrow caused the accumulation of Tregs in breast cancer tissue leading to both antitumor immunity and local immune suppression in breast cancer [55]. The mechanisms underlying pro-tumor role of Foxp3^+^ Tregs included (ⅰ) down-regulation of Notch pathway [56]; (ⅱ) direct suppression via cell-cell contact and indirect suppression via secretion of anti-inflammatory mediators such as interleukins (IL-4, IL-5 and IL-10) [57,58,59]; (ⅲ) decreased secretion of cytokine IFN-γ and IL-17 and activation of STAT1/STAT3 [59]. The prognostic significance of Tregs in TNBCs, therefore, remains controversial and warrants more careful investigations.

#### 2.1.3. M2 Macrophages

M2 macrophages, the main tumor-associated macrophages, (TAMs), can promote breast cancer initiation, angiogenesis, invasion, and metastasis by generating an immunosuppressive TME via releasing cytokines, chemokines, and growth factors [60]. TAMs expressing CD163^+^ (marker of M2 macrophages) positively correlate with tumor associated fibroblasts and epithelial-mesenchymal transition, which in turn are associated with aggressive behaviors and short DFS in 278 patients with histologically confirmed TNBC [61,62]. Another clinical study showed that high CD68^+^ (marker of M2 macrophage) TAMs expression associates with poor distant metastasis free survival (DMFS), DFS and OS in 287 patients with TNBC [63]. Mechanistically, in vivo and in vitro studies showed that the presence of CD11b^+^F4/80^+^CD206^+^ TAMs significantly associate with proliferating tumor cells in a TNBC mouse model. RNA sequencing analysis revealed that TAMs promote MAPK pathway activation in 4T1 cells [64]. Reactive oxygen species (ROS)-induced macrophages produce an immunosuppressive subtype (M2) and increase the expression of PD-L1 via activating NF-κB signaling, as well as release immunosuppressive chemokines such as interleukin-10 (IL-10), IL-17, IL-4, IL-1β, insulin-like growth factor-binding protein 3 (IGFBP-3), and chemokine (C-X-C motif) ligand 1 (CXCL1) [65]. The JAK2/STAT3 signaling pathway can up-regulate the expression of PD-L1 in CD169^+^ macrophages, but cannot up-regulate the expression of PD-L1 in breast cancer cells, thus avoiding immune surveillance [66]. Metastasis- and inflammation-associated microenvironmental factor S100A4 activates the basal-like subtype of breast cancer cells to trigger monocyte-to-macrophage (M2) differentiation and polarization, and elevates secretion of pro-inflammatory cytokines such as IL-8, IL-6, CXCL10, CCL2 and CCL5 [67]. Further, macrophage colony-stimulating factor (M-CSF), the main stimulator of macrophage migration, caused aggregation of M2 macrophages through an increased elongation of pseudopodia [68]. Inhibitors of differentiation (ID) 4 significantly associates with M2 macrophage marker CD68 protein expression in a series of TNBC tissues. ID4 activates an angiogenic procedure at the molecular level in the macrophages through paracrine signaling including the decrease of constituents of the anti-angiogenic miR-15b/107 group and an increase of angiogenesis-associated mRNAs [69,70]. GM-CSF BRCA1-IRIS overexpressing TNBC cells secrete high quantities of GM-CSF in an NF-κB and a HIF-1α-dependent manner to induce macrophages to IRIS overexpressing cells and polarize them to pro-tumor TAMs (M2). GM-CSF triggers TGF-β1 expression on TAMs through activating STAT5, NF-κB and/or ERK signaling [71].

#### 2.1.4. MDSCs

Myeloid-derived suppressor cells (MDSCs) are an important part of immunosuppressive network [72]. CD33^+^ MDSCs are a risk factor for progressive disease (PD) plus stable disease (SD) in breast cancer tissues prior to neoadjuvant chemotherapy [73]. Higher expression of MDSCs has been noted in TNBCs in comparison to non-TNBCs with their recruitment to the primary cancer and metastasis occurring via ΔNp63-dependent activation of the chemokines CCL22 and CXCL2 [74]. Glycolysis restriction reduces MDSCs through inhibiting cancer granulocyte G-CSF and GM-CSF expression [75] while hypoxia enhances the expansion of MDSCs and upregulates the expression of PD-L1 in the hypoxic TME of 4T1 tumor-bearing mice [76]. Studies have shown that the monoclonal antibody that neutralizes IL-8 (HuMax-IL8) and the traditional Chinese medicine Prim-O-glucosylcimifugin (POG) can inhibit the recruitment, proliferation, metabolism and immunosuppressive ability of MDSCs [77,78]. The 4T1 TNBC model effectively exhibits induction of immunosuppressive MDSCs accumulation by releasing inflammatory cytokines that produce permissive pro-metastatic TME [79]. Monocytic MDSCs (M-MDSC) and granulocytic MDSCs (G-MDSC) are two types of MDSCs in circulating peripheral blood. G-MDSC levels increase sharply and M-MDSCs decrease significantly after doxorubicin and cyclophosphamide treatment [80]. Investigations have shown that CCL5 is a key modulator of Rb1 activation and is associated with the immunosuppressive activity of MDSCs, especially the G-MDSC subset [81,82].

### 2.2. Immunoreactive TME in TNBC

#### 2.2.1. NK Cells

Natural killer (NK) cells, a type of cytotoxic lymphocytes, are crucial constituents of the innate immune system whose function in enhancing the anti-tumor immunity in TNBC has been studied extensively. NK cells are abundant in early cancer tissue in human solid tumors; however, they dwindle in metastatic human cancers [83]. These findings show that NK cells play a key role in immune surveillance, but once tumorigenesis occurs, TME is suppressive for NK cells. Evasion of active immune suppression in the TME is an important consideration for enhancing the anti-tumor ability of tumor-infiltrating NK cells. Zhang and colleagues detected the expression of NKp46, Foxp3, CD8, CD163 or Gas6 in 278 TNBC tissues using IHC with an aim to develop a prognostic risk model for TNBC. Multivariate analysis showed that TNM stage, Foxp3 positive lymphocytes along with prognostic risk scores can be used as independent indicators of OS and DFS in TNBC [84]. Tumor-derived IL-18 upregulates PD-1 expression on CD56^dim^CD16^dim/−^ NK cells and relates to the bad/ prognosis of TNBC [85]. McArdle and colleagues examined the abundance of NK cells, MDSCs, monocyte subsets and Foxp3^+^ Tregs in the peripheral blood of 85 breast cancer patients and they found that chemotherapy had no effect on the percentage of these immune cells, but peripheral blood cells could distinguish TNBC patients that are at high risk of relapse after chemotherapy [86]. Tissue-infiltrating NK cells in solid tumors appear to have a less robust activity compared with circulating NK cells [87,88,89,90]. NK cells isolated from either breast cancer patients or healthy donors show high cytotoxicity against patient-derived tumor cells in vitro and prevent tumor initiation and growth in immunocompromised mice in vivo [91]. Expanded cord blood-NK cells show cytotoxicity towards primary breast tumor cells derived from TNBC and estrogen receptor-positive/progesterone receptor-positive breast cancer [92]. Baseline circulating tumor cells (CTCs) status is positively associated with peripheral NK cells among those receiving first-line treatment in 75 patients with TNBC. Baseline CTCs combined with peripheral NK enumeration (CTC-NK) can predict PFS of TNBC patients more precisely [93]. NK cells are the major effectors of antibody (Ab)-dependent cell-mediated cytotoxicity (ADCC) and thus play an important role in Ab-based therapies. In vivo and in vitro studies revealed that tissue factor (TF)-targeting antibody-like immunoconjugate (called L-ICON)-CAR-NK cells have direct killing effects against TNBC cells and also mediate L-ICON ADCC to acquire a stronger effect [94]. Avelumab, a human IgG anti-PD-L1 mAb, triggers ADCC against a panel of TNBC cells and enhances NK-cell mediated cytotoxicity, which is independent of the blockade of the PD-1/PD-L1 pathway but is involved with IL-2 and IL-15 [95]. CD85j, an inhibitory receptor which can recognize both classical and non-classical HLA-I molecules, is highly expressed in TNBC, and can impair the function of cetuximab through NK-cell functional deficiency even when stimulatory cytokines IL-2 or IL-15 are abundantly present [96]. More interestingly, NK cell infiltration and recruitment can be mediated by a bispecific Ab (MesobsFab) whose anti-tumor activity depend on mesothelin expression on the target cells and it can be a potential antibody-based immunotherapeutic for TNBC patients [97]. NK cell function is regulated by molecules from promoting and suppressing receptors interacting with ligands on target cells. Lectin-like Transcript-1 (OCIL, CLEC2D, LLT1) is a ligand that interacts with NK cell receptor NKRP1A and prevents NK cell activation. Inhibiting LLT1 on TNBCs with antibodies hinders the interaction with NKRP1A and increases lysis of TNBCs by primary NK cells [98].

#### 2.2.2. CD8^+^ TILs

CD8^+^ TILs are the main kind of cytolytic lymphocytes in tumors. Kronqvist and group detected the expression of stromal TILs and CD8^+^ TILs in 179 patients with TNBC using IHC and observed that the prognostic value of CD8^+^ TILs and TILs varied when detected in various cancer compartments [99]. Presence of CD8^+^ TILs in a large cohort of 12,439 breast cancer patients correlated with a significant decrease in the relative hazard of death in both the ER- positive and the ER- negative HER2-positive subtypes [100]. Ishida and colleagues assessed the CD8^+^ TILs and Foxp3^+^ Tregs status of the residual tumors in 131 patients with TNBC who received neoadjuvant chemotherapy (NAC) at three institutions and the rates of their changes before and after NAC were evaluated. They found that TNBC patients with a high CD8^+^ TILs level or high CD8/Foxp3 ratio in residual tumors exhibit significantly favorable recurrence-free survival (RFS) and breast cancer-specific survival (BCSS) [101]. Another study also showed that CD8^+^ TILs were related to favorable DMFS, DFS, and BCSS in the entire 207 breast cancer group and in 56 TNBC group [102]. BRCA1-IRIS overexpressing (IRISOE) TNBC carcinomas had more CD25^+^/Foxp3^+^ Tregs and few CD8^+^/PD-1^+^ cytotoxic T-cells, which showed that the interaction between macrophages and IRISOE cells initiated an immunosuppressive TME within TNBC tumors [71]. TOPOIIα and CD4^+^ TILs were significantly positively associated with CD8^+^ TILs and they exhibited a significantly good 5-year DFS but only a high infiltration of CD8^+^ TILs showed significantly better 5-year OS in 52 TNBC patients that received taxane-anthracycline-based NAC [103,104]. Calcium/calmodulin-dependent kinase (CaMKK2), expressed in tumor-related stromal cells, could promote tumor growth. The inhibition of CaMKK2 within myeloid cells suppresses tumor growth by increasing immune-stimulatory myeloid subsets and intra-tumoral accumulation of CD8^+^ T cells in TNBC [105]. PARP inhibitor Olaparib induced CD8^+^ T cell activation and infiltration via activation of the cGAS/STING pathway, which provided rationale for combining the PARP inhibitors with immunotherapies for TNBC [106]. A recent study reported that CD8^+^ TILs were crucial for infected cell vaccine (ICV) efficacy, which was composed of autologous tumor cells infected with an oncolytic Maraba MG1 virus in vitro in the BALB/c-4T1 model. Increased migration and proliferation ability of human CD8^+^ TILs were observed following exposure to ICV [107]. A series of studies illuminated the mechanisms of different infiltration levels of CD8^+^ TILs in immunomodulation and anti-tumor response of TNBC. By spatially modulating the diffusion/chemotactic coefficients of T cells via partial differential equations, Almohanad et al. found that a type of chemorepellent inside cancer cell clusters but not dense collagen fibers, prevents the infiltration of CD8^+^ TILs into cancers and cancer cell clusters, which may imply a poor prognosis in TNBC [108]. Intra tumoral CD8^+^ TILs enhance the efficacy of treatment through triple combined inhibition of PDGFRβ/ MEK1/2/JAK2 signal pathway in vivo in TNBC [109]. Gruosso et al., found that there were many different kinds of CD8^+^ TILs localization profiles with distinct meta-signatures, which were prognostic indicators in a cohort of TNBC [17]. Dong et al. investigated the genome-scale CD8^+^ TILs CRISPR screen in the context of immunotherapy in vivo and in vitro and found that DHX37 interacts with PDCD11 and affects NF-κB activity to modulate CD8^+^ TILs activation, cytokine production, and cytotoxicity [110].

#### 2.2.3. M1 Macrophages

M1 phenotype macrophages, also called classical macrophages, are pro-inflammatory, and can activate the immune response and oppose tumorigenesis [111]. In vitro and in vivo studies have shown that M1 macrophage polarization decreases the expression of nuclear REST corepressor 1 (CoREST), LSD1 and the zinc finger protein SNAIL, and LSD1 inhibitors can target both CoREST and flavin adenine dinucleotide (FAD) binding domains of LSD1 to initiate macrophages toward M1 phenotype in TNBC successfully [112]. Another study revealed that exposure to infected cell vaccine (ICV) could induce the polarization of monocytes to M1 subtype [107].

Using the 4T1 TNBC murine model, Meyer and colleagues showed that in the early stages of disease, higher M1-related cytokines are released and decreased M2 macrophages infiltrate in the TME, while upon metastasis a dramatic enhancement in M2-related cytokine expression levels are detected and more immunosuppressive cells such as M2 macrophages infiltrate in the TME [113]. High level of CCL5 is related to recruitment of M1 macrophages, CD8^+^ TILs, CD4 activated T lymphocytes, and NK activated cells in TNBC using CIBERSORT analysis [114]. The clinical significance and involved mechanisms of each constituent in TNBC microenvironment are included in Table 1.

**Table 1:** Clinical significance and involved mechanisms of immune cells and markers.

Items	Clinical Significance	Involved Mechanisms	References
PD-1/PD-L1	Paradoxical role in prognosis	microRNA-200/ZEB1 axis, WNT signaling, loss of PTEN, PI3K signaling, and MUC1-C/MYC/NF-κB pathway	[31,34,35,36,37,38,39,40,41,44,45,46]
Foxp3+ Tregs	Paradoxical role in prognosis	Notch pathway, IL-35/STAT1/STAT3, secretion of anti-inflammatory mediators such as interleukin	[50,51,52,53,54,56,57,58,59,115]
M2 macrophages	Adverse prognostic indicator	MAPK pathway, NF-κB/PD-L1, release of immunosuppressive chemokines, JAK2/STAT3 signaling pathway, S100A4 activation, angiogenic program, HIF-1α, STAT5, NF-κB and ERK signaling	[61,62,63,64,116]
MDSCs	Risk factor for PD plus SD	ΔNp63-dependent activation of the chemokines CXCL2 and CCL22, Glycolysis, hypoxia, secretion of inflammatory cytokines, Rb1 activation	[73,74,75,76,81,82]
NK cells	Positive prognostic indicator	ADCC, Lectin-like Transcript-1 activation, bispecific antibody (MesobsFab) modulating chemorepellent inside tumor cell clusters	[84,85,92,94,95,96,97,98,117]
CD8^+^ TILs	Favorable prognostic indicator	Inhibition of PDGFRβ/MEK1/2/JAK2 signal pathway, distinct metasignatures of CD8+ TILs, DHX37/PDCD11/NF-κB	[17,99,100,101,108,109,110]
M1 macrophages	Favorable prognostic indicator	M1 polarization by FAD, CoREST and exposure to cell vaccine (ICV), release of CCL5	[112,113,114]

## 3. The Composition of TME Contributes to TNBC Subtype Classification

During TNBC progression, TME reconstruction including the ratio of immune cells and release of various immune cytokines play crucial roles, and the research focusing on stromal and immune composition of TME has contributed significantly to different subtype classification of TNBC [17]. Lehmann and colleagues distinguished six TNBC subtypes showing unique gene expression profiles and ontologies, comprised of two basal-like (BL1 and BL2), a mesenchymal stem-like (MSL), a mesenchymal (M), an immunomodulatory (IM), and a luminal androgen receptor (LAR) subtype. Interestingly, immune genes in IM subtype overlap with gene signatures in medullary breast cancer which is correlated with good prognosis despite its high-grade scores [118]. Park and colleagues distinguished four stromal axes abundant for T cells, B cells, epithelial markers and desmoplasia and assigned a score along with each marker and associated it with different TNBC subtypes. This classification method better depicted tumor heterogeneity and led to a superior evaluation of benefit from therapeutics and prognosis [119].

In addition, three subtypes of TNBC have been identified: an apocrine cluster (C1), which is more related to luminal, PIK3CA-mutated hallmarks and shows intermediate biological aggressiveness; and two basal-like clusters (C2 and C3), which show a major biological discrepancy related to immune response and are sensitive to drugs combating immunosuppression or stimulate adaptive immune response respectively [120]. Shao and colleagues analyzed genomic, clinical, and transcriptomic data of 465 primary TNBC patients, and also identified four subtypes of TNBC, including basal-like immune-suppressed (BLIS), immunomodulatory (IM), luminal androgen receptor (LAR) and mesenchymal-like (MES). They also showed that IM subtype is related to immune response and there are elevated immune cell signaling, TILs, high mRNA expression quantities of immune checkpoint blocking genes such as PD-L1, PD-1, CTLA4, and IDO1 [121]. Using the data of 465 Taiwanese with breast cancer, five TNBC subtypes were classified, namely, basal-like (BL), mesenchymal stem like (MSL), immunomodulatory (IM), mesenchymal (M), and luminal androgen receptor (LAR), and they observed the interaction between IM subtype and MSL subtype, which also implied the involvement of TME in TNBC subtype classification [122]. Distinguishing a four-gene decision tree signature (*TP53BP2, EXO1, RSU1* and *FOXM1*) using transcriptomic and genomic data analysis established six subtypes of TNBC, named MC1 to MC6, comprised by five of varying sizes (MC1-MC5) and one large subtype MC6. Further study showed high level of CD8^+^ and CD4^+^ immune signatures and decreased expression of MAPK pathway related genes in MC6 subtype [123]. Another group identified three TNBC subtypes including Immunity High (Immunity H), Immunity Medium (Immunity M), and Immunity Low (Immunity L) based on the immunogenomic profiling of 29 immune signatures. In Immunity H subtype, greater anti-tumor immune response and immune cell infiltration, as well as favorable prognosis were detected compared to the other subtypes, which showed the close relationship between tumor immune microenvironment and TNBC classification [124]. TNBC tumors were classified into four subgroups (luminal-androgen receptor expressing, basal, claudin-high and claudin-low), in addition to two subgroups associated with immune activity using gene expression and clinical data and the latter two immune subgroups were defined as correlated to immune activity closely. Meanwhile, claudin-high subgroup had low response to neoadjuvant chemotherapy, and luminal immune-positive subgroup had favorable survival prognoses [125]. A recent study identified four TNBC epitopes, named as Epi-CL-A, Epi-CL-B, Epi-CLC, and Epi-CL-D using genome-wide DNA methylation properties and clinical and demographic variables, as well as gene mutation and gene expression data. Intriguingly, subtype Epi-CL-D showed a positive regulation of T lymphocyte-mediated cytotoxicity and associated molecules such as IL15RA and CCL18, which partially explained the favorable outcome and a positive immune response in this subtype [126]. Furthermore, a research group classified TNBC tumors into immune subtype A and B by the density of monocytes, γδ T cells, stromal CD4^+^ T cells, M1 macrophages and M2 macrophages using CIBERSORT or IHC method and they proved that enriched immune-related pathways and higher levels of immune checkpoint cytokines such as PD-1, PD-L1 and CTLA-4 could be detected in phenotype A [127]. Romero-Cordoba et al. also identified three immuno-clusters in TNBC tumors using clustering analysis based on immune-related gene expression signatures and found that platelet to lymphocyte ratio (PLR) was associated with tumor immune infiltration [128].

We have included all the classification methods and the clinical significance (Table 2). Classification of TNBC has been developed extensively implying that a precision-treatment era has come in TNBC. Chemotherapy still remains the key treatment for TNBC but other targeted therapies including immunotherapy can be combined for better tailored treatments and are the focus of ongoing research efforts.

**Table 2:** TNBC subtype classification.

Subtype of TNBC	Subtype Number	Basis of Classification	Clinical Significance	References
BL1, BL2, IM, M, MSL, LAR	6	Gene expression profiles	IM subtype was associated with favorable prognosis.	[118]
4 stroma axes (T,B,E,D)	4	Transcriptome of stroma	Better evaluated patient benefit from therapeutics.	[119]
C1, C2, C3	3	Gene expression profiling	C2 and C3 subtypes were sensitive to drugs combating immunosuppression.	[120]
LAR, IM, BLIS, MES	4	Clinical, genomic, and transcriptomic data	Elevated immune cells and signaling in IM subtype.	[121]
BL, IM, M, MSL, LAR	5	Gene expression profiles	Interaction between IM and MSL subtype suggested involvement of TME.	[122]
MC1, MC2, MC3, MC4, MC5, MC6	6	Transcriptomic and genomic data	High level of CD8^+^ and CD4^+^ immune signatures in MC6 subtype.	[123]
Immunity_H, Immunity_M, Immunity_L	3	Immunogenomic profiling	Immunity_H subtype was correlated with immune cell expression and good prognosis	[124]
LAR, basal, claudin-low, claudin-high and two immune subtypes	6	Clinical and gene expression data	Claudin-h and immune-positive subtype was associated with low pCR and favorable prognosis separately.	[125]
Epi-CL-A, Epi-CL-B, Epi-CLC, Epi-CL-D	4	Genome-wide DNA methylation profiles	Positive regulation of T lymphocyte cytotoxicity and associated genes in Epi-CL-D subtype.	[126]
Immune phenotype A and B	2	Density of five prognosis-related immune cells	Enriched immune-related pathways and molecules in phenotype A.	[127]
ImA, ImB and ImC	3	Immune-related gene expression signatures	Platelet to lymphocyte ratio (PLR) was associated with tumor immune infiltration in TNBC.	[128]

## 4. Chemotherapy-Induced TME Remodeling Modulates TNBC Immune Response

**Figure 2:**
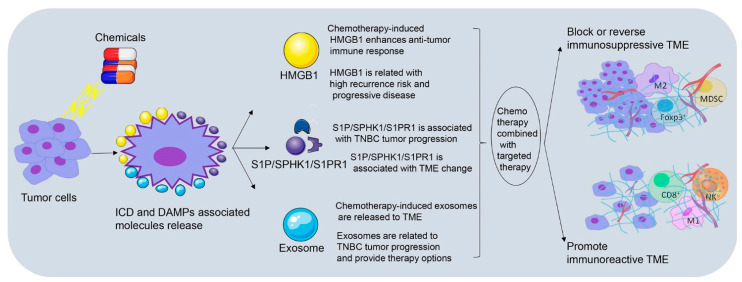
Chemotherapy-induced immunogenic cell death and immunomodulation in TNBC. Chemotherapy induces immunogenic cell death (ICD), and then promotes the release of damage-associated molecular patterns (DAMPs) including high mobility group box1 (HMGB1), exosomes and sphingosine-1-phosphate receptor 1 (S1PR1) by damaged or activated cells. Chemotherapy combined with targeted therapy could enhance anti-tumor immunity through promoting function of immunoreactive lymphocytes and blocking or reversing function of immunosuppressive cells. (ICD, immunogenic cell death; DAMPs, damage-associated molecular patterns; HMGB1, high mobility group box1; S1P, sphingosine-1-phosphate; SPHK1, sphingosine kinase 1; S1PR1, sphingosine-1-phosphate receptor 1; TNBC, triple negative breast cancer; TME, tumor microenvironment).

It has been reported that cytotoxic drugs such as anthracycline and platinum agents, could induce immunogenic cell death (ICD), and stimulate anti-tumor immune response of T lymphocytes [18,129]. Damage-associated molecular patterns (DAMPs) are cytokines that are released by damaged or activated cells; have great immune stimulating response, and cause ICD [18]. ICD involves the cell surface exposure of calreticulin (CRT), release of DAMPs-related high mobility group box1 (HMGB1) and autophagy-dependent ATP release, which together, leads to the antigen uptake and presentation of DC cell, and then activates the CD8^+^ TILs to play the anti-tumor role [130,131]. Carboplatin or paclitaxel combined with radiation generates both chemotherapeutic enhancement of ICD and a dose-dependent induction of ICD in TSA mammary carcinoma cells [132]. Doxorubicin and paclitaxel treatment results in the recruitment of innate immune cells and CSF1R-dependent macrophages infiltration in PyMT-MMTV mammary carcinoma through an increase of CCL2, CXCL2, CSF-1, interleukin-34 and vascular permeability [133,134]. Docetaxel polarizes MDSCs toward M1-like phenotype and upregulates macrophages markers (CD86, MHC class II, and CD11c) in vivo and in vitro partly through an inhibition of STAT-3 in 4T1-Neu mammary cancer implants [135]. All these studies emphasize that chemotherapy can induce TME remodeling through distinct signaling pathways. In this part, we have focused on three crucial factors related to chemotherapy-induced TME remodeling, which are HMGB1, exosomes and S1PR1. The clinical significance of HMGB1, exosomes and S1P/SPHK1/S1PR1 as well as their involvement in TNBC immunomodulation and tumor progression is shown in Figure 2.

### 4.1. Chemotherapy-Induced HMGB1 Release Participates in TNBC Immunomodulation

#### 4.1.1. Chemotherapy-Induced HMGB1 Enhances Anti-Tumor Immune Response

High mobility group box1 (HMGB1) is a highly conserved DNA-binding nuclear protein, involved in many kinds of diseases, including cancer, arthritis, and sepsis [136]. Extracellular HMGB1 in response to inflammation activates the host immune system. HMGB1 can combine with TLR-2, TLR-4, and TLR-9, and recruit the inflammatory cells to microenvironment. This activates the DCs, enhances the antigen presentation ability and anti-tumor immune response [137].

#### 4.1.2. HMGB1 Is Related to High Recurrence Risk and Progressive Disease after Neoadjuvant Chemotherapy

A study indicated that the nuclear expression of HMGB1 in breast cancer cells negatively correlates with Tregs and TAMs [138], and could predict the recurrence risk of residual tumor [139]. HMGB1 expression in cytoplasm is higher in HER2-positive and TNBCs tumors than in hormone receptor (HR)-positive tumors. High cytoplasmic HMGB1 significantly correlates with advanced histologic grade, abundant TILs, and high expression of CD8^+^ TILs but shows no prognostic significance in TNBC [140]. Intracellular HMGB1 expression has been detected in fibroblasts conditioned medium (CM) treated breast cancer cells and in doxorubicin-treated cells. Extracellular HMGB1 is upregulated in CM after doxorubicin-induced MDA-MB-231 cell death, which show the potential of fibroblasts in stroma to contribute to chemo-resistance partly by fibroblast-induced HMGB1 production [141]. It has been shown that low cytoplasmic HMGB1-positive breast tumor cells and high ASMA-positive fibroblasts predict adverse prognosis in TNBC [142]. Tanabe and colleagues reported that positive HMGB1 expressions are higher in the clinical progressive disease (cPD) than in control group during neoadjuvant chemotherapy in TNBC patients [143]. Some of HMGB1 single nucleotide polymorphisms (SNPs) have been related to tumor progression in T2 tumor, pathologic grade 3 disease, and distant metastasis in TNBC and HER2-enriched tumors compared with luminal tumors [144]. By targeting HMGB1-RAGE signaling pathway, miR-205 impairs the viability and epithelial-to-mesenchymal transition in TNBC cells [145]. HMGB1 released by breast cancer cells is N-glycosylated at Asn37, which promotes the transition from monocytes to MDSC-like cells and contributes to M-MDSC differentiation from bone marrow through the p38/NFκB/Erk1/2 signaling pathway [146].

### 4.2. Chemotherapy-Induced Exosomes Secretion Interconnects TME and TNBC Immune Response

#### 4.2.1. Chemotherapy-Induced Exosomes Are Released to TME

Exosomes are tiny membrane vesicles (30–100 nm in diameter) synthesized in late endosomes and secreted into the extracellular milieu by various cells. They contain functional molecules (lipids, proteins, DNA, and RNA) that can be transferred to recipient cells, playing a key role in intercellular communication [147]. Apoptosis exosome vesicles (AEVs) are special exosomes overexpressing S1PR1 and S1PR3 released by the tumor cells in response to certain chemicals. These AEVs induce the expression of inflammatory chemokines and cytokines which participate in the pathological and physiological process of DAMPs [147].

#### 4.2.2. Exosomes Are Related to TNBC Tumor Progression and Provide Therapy Options

Some investigations have explored connections between exosomes and TNBCs [148]. Hypoxia induces the production of exosomes and microvesicles (MVs) in breast cancer cells through HIF-dependent RAB22A expression, which can stimulate ECM invasion, focal adhesion formation, lung colonization and is associated with decreased OS and MFS in the mouse models [149]. Stevic and colleagues determined miRNA expression profiles of exosomes originated from the plasma of TNBC and HER2-positive breast cancer patients before neoadjuvant therapy. They found that exosomal miRNAs (miR-155 and miR-301) correlate with the risk factors and clinicopathological factors significantly and can predict pCR rate [150]. Extracellular vesicles (EVs) from HCC1806 but not from MDA-MB-231 cells exhibit enhanced drug resistance and alter the levels of genes involved in cell apoptosis and proliferation pathways in MCF10A cells [151]. Ni and colleagues quantified the levels of miRNAs expression in exosomes from plasma of 8 ductal carcinoma in situ (DCIS) patients, 32 breast cancer (BC) patients and 8 healthy women; they found that different levels of exosomal miRNAs had distinct prognostic value in different subtypes of BC and the expression of miR-16 was lower in TNBC than HR-positive counterparts [152]. Exosomes from TNBC tissues regulate cell apoptosis and TME changes. MiR-770 played its multi-functional role in TNBC by down-regulating gene STMN1 as follows: (ⅰ) was associated with favorable prognosis of TNBC, (ⅱ) increased the sensitivity of TNBC cells to doxorubicin through induction of apoptosis, (ⅲ) regulated TAMs-induced chemotherapy resistance, and (ⅳ) inhibited invasion and migration ability of TNBC cells via EMT pathway [153]. Intriguingly, chemotherapy-induced senescent cells secreted more extracellular vesicles than non-senescent cells in TNBC [154]. Exosomes could facilitate co-delivery of cholesterol-modified miR-159 and therapeutic quantities of doxorubicin to TNBC cells both in vitro and in vivo [155]. A formulation of erastin (a low molecular weight chemotherapy drug that induces ferroptosis)-loaded exosome was labeled with special chemicals to target TNBC cells, which enhanced the uptake efficiency of drugs into MDA-MB-231 cells and had a better preventing effect on the migration and proliferation, revealing that the exosome-based therapy might serve as a novel and powerful delivery method for anti-cancer therapy [156].

### 4.3. S1P/SPHK1/S1PR1 Link TME Changes to TNBC Immunomodulation

#### 4.3.1. S1P/SPHK1/S1PR1 Is Associated with TME Changes

Sphingosine-1-phosphate (S1P), a novel lipid signaling mediator with both intracellular and extracellular functions, is generated by sphingosine kinase 1 (SPHK1), an enzyme catalyzing phosphorylation of sphingosine. S1P/SPHK1 interacts with constituents in TME and modulate the progression and metastasis of breast cancer. Binding of S1P to sphingosine-1-phosphate receptor (S1PRs) on cell surface activates cytokines in the cytoplasm and gene activation in the nucleus in an autocrine and paracrine manner [157,158]. S1P, S1PRs, and SPHK1 expression are related to metastatic progression in breast cancers in vivo [159]. An investigation in melanoma suggested that S1PR1 causes immune functional change of T lymphocytes via PPARγ signal pathway [160]. A recent investigation in breast cancer showed that S1PR1 causes the change of TAMs phenotype, promotes neo-lymph vascularization, and the change of TME via activating inflammatory factors such as Nlrp3 and IL-1β [161]. Another team also showed that S1PR1 phosphorylates the complex of vasculogenic mimicry (VM), and the inhibition of S1PR1 decreases endothelium-dependent vessel (EDV), but causes the production of VM, invasion, and metastasis in vitro and in vivo [162]. Kim and colleagues showed that IL-22 induces S1PR1 and IL22R1 expression in myeloid cells and macrophages, and induce MCP1 expression in myeloid stem cells (MSCs), and then facilitate macrophage infiltration, implying a potential effect of IL-22 on promoting bone metastasis of breast cancers via IL22R1/S1PR1 pathway [158]. S1P1 is expressed in tumor antigen-specific bone marrow (BM) Tregs selectively in breast cancer, and can be induced by BM-resident antigen-presenting cells in conjunction with T cell receptor stimulation [163].

#### 4.3.2. S1P/SPHK1/S1PR1 Is Associated with TNBC Tumor Progression

A preclinical study detected the function of S1PR1-antibody on the growth of breast cancer cell lines MDA-MB-231 and SK-BR-3. They found that S1PR1-antibody not only increases the cytotoxicity of carboplatin on MDA-MB-231 cells but also enhances the anti-proliferative outcome of S1P on SK-BR-3 cells [164]. It has been reported that apoptotic tumor cells release S1P, and then stimulate the generation of lipocalin 2 (LCN2) in TAMs and is associated with breast cancer metastasis [165]. As the key kinase of S1P combination, SPHK1 has been found to be overexpressed in TNBC compared with other breast cancer subtypes, and promotes tumor metastasis. By targeting SPHK1 or its downstream signaling pathway (NF-κB pathway) with available inhibitors, TNBC metastasis is effectively inhibited [166]. Maiti and colleagues found that SPHKs/S1P axis is a crucial constituent of survival and growth of LM2-4 cells compared to parental MDA-MB-231 cells, and nuclear SPHK2 (in MDA-MB-231 cells) is also indispensable for LM2-4 cells survival and growth [167]. Obesity and high-fat diet are the main cause for increased expression of the S1P and SPHK1, and targeting the SPHK1/S1P/S1PR1 decreases key proinflammatory cytokines, macrophage infiltration, and tumor progression [168]. However, Lei and colleagues found that S1PRs expression inhibits tumor progression in breast cancer patients [169]. The clinical significance of DAMPs-associated molecules (HMGB1, exosomes, and S1P/SPHK1/S1PR1) and the mechanisms involved in TNBC immunomodulation and tumor progression are included in Table 3.

**Table 3:** Clinical significance and involved mechanisms of DAMPs-associated molecules.

Items	Clinical Significance	Involved Mechanisms	References
HMGB1	Predict recurrence risk of residual tumor after neoadjuvant chemotherapy	TLR4 signal pathway, immune molecules such as TGF-β, IK12p7, and IFN-γ, p38/NFκB/Erk1/2 pathway, RAGE/IRF3/NF-κB	[19,20,139,140,142,144,145,146,170]
Exosome	pCR prediction and distinct prognosis value in different subtype of breast cancer	HMGB1/TLR4/NF-κB signaling	[150,152,171,172]
S1P/SPHK1/S1PR1	Paradoxical role in tumor progression of TNBC	PPARγ signal pathway, STAT3/IL-6, IL-22, TCR activation	[158,160,163,169,173,174,175]

## 5. Conclusions

The role of the tumor microenvironment (TME) in triple negative breast cancer (TNBC) immunomodulation is vitally important. The deeper understanding of immunosuppressive and immunoreactive TME has contributed to specific subtype classification of TNBC. In future, we may be able to treat TNBC patients with more precision according to their subtype. Agents that remodel TME, promote function of immunoreactive lymphocytes, block function of immunosuppressive cells, and prevent inhibitory signaling pathways can all be considered. Furthermore, therapies targeting HMGB1, exosomal microRNAs, and S1P/SPHK1/S1PR1, can also be considered in combination with chemotherapy. In conclusion, immunosuppressive and immunoreactive role of TME, the contribution of TME in TNBC subtype classification, chemotherapy-induced TME changes and its role in TNBC immunomodulation are crucial for TNBC management. TME has provided a new direction to explore novel and effective combination regimens for precision treatment of TNBC.
